# Genetic variation in the methylenetetrahydrofolate reductase gene, *MTHFR*, does not alter the risk of visual failure in Leber’s hereditary optic neuropathy

**Published:** 2009-05-01

**Authors:** Gavin Hudson, Patrick Yu-Wai-Man, Massimo Zeviani, Patrick F. Chinnery

**Affiliations:** 1 Mitochondrial Research Group, Institute of Ageing and Health, Newcastle University, Newcastle upon Tyne, United Kingdom; 2 Unit of Molecular Neurogenetics, Pierfranco and Luisa Mariani Center for the Study of Children's Mitochondrial Disorders, Foundation “C. Besta” Neurological Institute-IRCCS, Milan, Italy

## Abstract

**Purpose:**

Focal neurodegeneration of the optic nerve in Leber hereditary optic neuropathy (LHON) is primarily due to a maternally inherited mitochondrial DNA mutation. However, the markedly reduced penetrance of LHON and segregation pattern of visual failure within families implicates an interacting nuclear genetic locus modulating the phenotype. Folate deficiency is known to cause bilateral optic neuropathy, and defects of folate metabolism have been associated with nonarteritic ischemic optic neuropathy.

**Methods:**

Methylenetetrahydrofolate reductase (*MTHFR*) catalyzes a critical step in folate metabolism, and genetic variation in *MTHFR* has been associated with several late-onset neurodegenerative diseases.

**Results:**

We therefore determined whether functional genetic variants in *MTHFR* could account for the reduced penetrance in LHON by studying 414 LHON mtDNA mutation carriers. We found no evidence of association between visual failure in LHON and *MTHFR* polymorphisms or the *MTHFR* haplotype.

**Conclusions:**

Genetic variation in *MTHFR* does not provide an explanation for the variable phenotype in LHON.

## Introduction

Leber hereditary optic neuropathy (LHON; OMIM #535000) is a common cause of inherited blindness that typically presents with bilateral, painless, subacute vision failure in young adult males. Affected individuals develop focal degeneration of the optic nerve and present clinically with impaired color vision (dyschromatopsia), a dense visual field defect (central or cecocentral scotoma), and abnormal visual electrophysiology due to primary retinal ganglion cell loss [1]. The diagnosis is usually confirmed by molecular genetic analysis for one of three common mitochondrial DNA (mtDNA) mutations which all affect genes coding for complex I subunits of the respiratory chain: m.3460G>A, m.11778G>A, and m14484T>C. However, only a few patients harboring a pathogenic LHON mtDNA mutation develop visual failure [2,3]. Segregation analysis of LHON pedigrees indicated a two-locus model: a mtDNA mutation as one locus and a modulating X-chromosomal locus [4]. Although an interacting X-chromosomal locus could explain the gender bias in LHON, not all pedigrees with LHON show linkage to the X-chromosome [5-7], and the segregation pattern in some pedigrees implicates one or more autosomal loci [8]. However, attempts to identify a nuclear modifying gene by both genetic mapping and functional genomics have so far failed to identify the interacting nuclear genes.

Folate is a necessary component for cellular maintenance and growth, especially important during early embryonic development, where it is involved in DNA synthesis. Methylenetetrahydrofolate reductase (*MTHFR*) catalyzes the conversion of 5,10-methylenetetrahydrofolate to 5-methyltetrahydrofolate, a critical step in the remethylation of homocysteine (Hcy) to methionine. Genetic variants in *MTHFR* are associated with hyperhomocysteinemia and cardiovascular disease [9] and are also associated with neural tube defects in the fetus [10]. c.677C>T, present at approximately 33%–37% heterozygously and roughly 10% homozygously in Europeans, leads to a substitution of alanine to valine (at position 222) in the catalytic domain of MTHFR, and subsequent reduction in enzyme activity [11]. This effect is magnified when c.677C>T is found as a compound heterozygote with homozygous c.1298A>C [12,13].

Previous studies have shown a link between oxidative stress and increased Hcy in neurodegenerative disorders [14,15], with a pronounced increase in Hcy in homozygote c.677C>T Alzheimer disease [16] and Parkinson disease [17]. Elevated levels of Hcy have been shown to cause endothelial dysfunction by increasing oxidative stress or impairing nitric oxide metabolism [18,19]. Increased Hcy was shown to induce apoptotic death in retinal ganglion cells, hypothesized as a cause of LHON [20], by overstimulation of the N-methyl-D-aspartate receptors and caspase-3 activation [21].

Increased Hcy, but not the c.677C>T variation, was identified as a risk factor in nonarteritic ischemic optic neuropathy and central retinal vein occlusion [22,23]. Folate deficiency is known to cause bilateral optic neuropathy [24,25]. Evidence is accumulating that implicates folate metabolism in optic neuropathies, particularly those affecting the retinal ganglion cell, making *MTHFR* a strong autosomal candidate genetic modifier in LHON, despite not localizing to the X chromosome and therefore less likely to contribute directly to the gender bias in LHON.

## Methods

We studied 12 common nonsynonymous *MTHFR* (NM_005957.3) single nucleotide polymorphisms (SNPs): (rs2066472, rs45550133, rs45438591, rs45571736, rs45496998, rs45449298, rs2274974, rs45590836, rs2274976, rs35737219, rs1801133, and rs1801131) in a European cohort of 414 LHON mtDNA mutation carriers (182 affected, 232 unaffected). All subjects were recruited from two European centers with local ethical review board approval in accordance with the declaration of Helsinki. 70% of the attached individuals were male, and 41% of the unaffected individuals were male, in keeping with the gender bias that characterizes LHON. All were homoplasmic for m.3460G>A, m.11778G>A, or m14484T>C. rs1801133 corresponds to c.677C>T, and rs1801131 corresponds to c.1298A>C. The additional ten SNPs were selected using the following criteria: 1) nonsynonymous substitutions predicted to affect *MTHFR* function; and 2) present in control subjects at >0.1% (dbSNP) [26].

The clinical phenotype was determined by a local ophthalmologist [1] and the genetic diagnosis was confirmed in affected individuals by mtDNA direct sequencing of the *MTND* genes or PCR-RFLP analysis. Control participants (unaffected mutation carriers) had no visual symptoms and were older (>30 years) than the median age of onset for LHON (24 years). The frequency of sequence variants was determined in European controls by primer extension of multiplex polymerase chain reaction products with the detection of the allele-specific extension products by matrix-associated laser desorption/ionization time of flight (MALDITOF; Sequenom, San Diego, CA) mass spectrometry. Genotype and allelic associations were compared using SPSS v15.0 using Fishers exact test. The p values given are two-tailed. To correct for multiple testing bias, we performed permutation testing using Haploview 4.0. Statistical power calculation is available at DSS research.

## Results

We analyzed SNP frequencies in 12 non-synonymous SNPs in a large LHON cohort. Six of the SNPs (rs2066472, rs45550133, rs45496998, rs45449298, rs2274974, and rs45590836) showed no variation and were removed from any further analysis. When males and females were considered together, Fisher exact test revealed a weak association between rs2274976 and LHON (Table 1, p=0.030). When males and females were considered separately (Table 2), rs35737219 was associated with visual failure in male LHON patients (p=0.043), When different LHON mutations were studied separately, we observed a significant association between rs45571736 and both m.3460 G>A and m.14484T>C (Table 3, p=0.018 and 0.028 respectively). However none of these associations were significant after correcting for multiple testing bias using permutation testing.

**Table 1:** Non-synonymous *MTHFR* variants in LHON.

**Genotype frequency**	**Patients**	**WT**	**Het**	**MT**	**P**
rs45438591	A	173	8	0	0.323
	C	222	6	0	
rs1801133	A	113	56	13	0.479
	C	151	60	21	
rs45571736	A	127	50	3	0.585
	C	172	54	3	
rs1801131	A	81	78	23	0.143
	C	112	102	16	
rs2274976	A	151	25	5	0.104
	C	207	19	3	
rs35737219	A	177	2	3	0.171
	C	221	9	2	
**Allele frequency**	**Patients**	**WT**	**MT**	**P**	
rs45438591	A	354	8	0.2854	
	C	450	6		
rs1801133	A	282	82	0.867	
	C	362	102		
rs45571736	A	304	56	0.364	
	C	398	60		
rs1801131	A	240	124	0.131	
	C	326	134		
rs2274976	A	327	35	0.03	
	C	433	25		
rs35737219	A	356	8	0.66	
	C	451	13		

Comparison of *MTHFR* variant genotype and allele frequencies between LHON patients (A) and controls (C; where WT and MT are homozygous wild-type and mutant, respectively and Het is heterozygous. P is an uncorrected Pearson’s chi-square probability).

**Table 2:** Gender specific *MTHFR* variants in LHON.

rs1801131 **:rs1801133**	**Total**	**A**	**U**	**P**
AA:CC	129	50	79	0.137
AA:CT	54	24	30	1
AA:TT	12	7	5	0.381
CA:CC	109	48	61	1
CA:CT	52	26	26	0.372
CA:TT	19	4	15	0.056
CC:CC	26	15	11	0.157
CC:CT	10	6	4	0.345
CC:TT	3	2	1	0.584
Total	414	182	232	

Comparison of c.677C>T and c.1298A>C (rs1801133:rs1801131) compound genotypes between LHON patients (A) and controls (C). P is an uncorrected Pearson’s chi-square probability).

**Table 3:** LHON mutation specific *MTHFR* variation

**Allele**		**3460**				**11778**			**14484**			**Other**		
		**A**	**C**	**P**	**A**		**C**	**P**	**A**	**C**	**P**	**A**	**C**	**P**
rs45438591	WT	32	47	0.231	118		149	0.333	8	8	1	12	16	0.393
	HET	1	0		6		4		1	1		0	1	
	MT	0	0		0		0		0	0		0	0	
rs1801133	WT	24	33	0.224	74		104	0.199	7	7	1	8	7	0.082
	HET	10	12		43		38		2	2		1	8	
	MT	0	4		10		15		0	0		3	2	
rs45571736	WT	31	34	0.018	83		118	0.166	1	6	0.028	12	14	0.124
	HET	3	15		41		35		6	1		0	3	
	MT	0	0		1		1		2	2		0	0	
rs1801131	WT	15	26	0.395	58		71	0.365	5	5	1	3	10	0.129
	HET	14	20		52		71		4	4		8	7	
	MT	2	3		17		13		0	0		1	0	
rs2274976	WT	27	41	0.624	111		143	0.13	4	7	0.148	9	16	0.141
	HET	5	6		15		10		2	2		3	1	
	MT	2	1		0		2		0	3		0	0	
rs35737219	WT	32	48	0.356	124		149	0.383	9	8	0.303	12	16	0.393
	HET	0	0		2		7		0	1		0	1	
	MT	2	1		1		1		0	0		0	0	
**Genotype**														
rs45438591	WT	65	94	0.413	242		302	0.355	17	17	1	24	33	1
	MT	1	0		6		4		1	1		0	1	
rs1801133	WT	58	78	0.415	191		246	0.423	16	16	1	17	22	0.778
	MT	10	20		63		68		2	2		7	12	
rs45571736	WT	65	83	0.04	207		271	0.09	8	13	0.176	24	31	0.26
	MT	3	15		43		37		10	5		0	3	
rs1801131	WT	44	72	0.856	168		213	0.528	14	14	1	14	27	0.142
	MT	18	26		86		97		4	4		10	7	
rs2274976	WT	59	88	0.436	237		296	0.451	10	16	0.438	21	33	0.297
	MT	9	8		15		14		2	8		3	1	
rs35737219	WT	64	96	0.228	250		305	0.402	18	17	1	24	33	1
	MT	4	2		4		9		0	1		0	1	

Comparison of LHON mutation specific *MTHFR* variant genotype and allele frequencies between LHON patients (A) and controls (C) where WT and MT are homozygous wild-type and mutant respectively and Het is heterozygous. P is an uncorrected Pearson’s chi-square probability.

When combining complex SNPs into complex genotypes we found no association to a compound genotype of c.677C>T and c.1298A>C (rs1801133:rs1801131; Table 4). We also performed six locus haplotyping analysis. There was no significant difference in the frequency of each genotype between affected and unaffected.

**Table 4:** c.677C>T and c.1298A>C complex genotypes in LHON

rs1801131 **:rs1801133**	**Total**	**A**	**U**	**P**
AA:CC	130	50	79	0.137
AA:CT	54	24	30	1
AA:TT	12	7	5	0.381
CA:CC	109	48	61	1
CA:CT	52	26	26	0.372
CA:TT	19	4	15	0.056
CC:CC	26	15	11	0.157
CC:CT	10	6	4	0.345
CC:TT	3	2	1	0.584
Total	415	182	232	

Comparison of c.677C>T and c.1298A>C (rs1801133:rs1801131) compound genotypes between LHON patients (A) and controls (C). P is an uncorrected Pearson’s chi-square probability).

## Discussion

We found no statistically robust association between any of the 12 functional *MTHFR* SNPs, individually or as complex genotypes, and vision failure in LHON families, or when affected individuals were compared to controls. It is intriguing that specific SNPs appeared to be associated with vision failure when considered in subgroup analyses separating the different LHON mtDNA mutations and different genders, but these associations did not stand up to the rigors of a correction for multiple significance testing. We therefore interpreted our findings conservatively, but larger studies may show that these associations are pathophysiologically relevant. Although we cannot exclude the possibility that *MTHFR* contributes to the pathophysiology of LHON, our findings indicate that the gene is unlikely to be the major nuclear genetic modifier interacting with the primary mtDNA mutations. Genes encoding other enzymes involved in folate metabolism may be relevant, as could dietary intake of folate. Biochemical and epidemiological studies would address these issues. Further genetic studies on a genome-wide level are required to define the nuclear-mitochondrial interaction in LHON.
